# The structural basis of eukaryotic chaperonin TRiC/CCT: Action and folding

**DOI:** 10.1016/j.mocell.2024.100012

**Published:** 2024-01-26

**Authors:** Hyunmin Kim, Junsun Park, Soung-Hun Roh

**Affiliations:** Department of Biological Sciences, Institute of Molecular Biology and Genetics, Seoul National University, Seoul 08826, Republic of Korea

**Keywords:** Chaperonin, TRiC/CCT, Protein folding, Structural biology

## Abstract

Accurate folding of proteins in living cells often requires the cooperative support of molecular chaperones. Eukaryotic group II chaperonin *T*ailless complex polypeptide 1-*Ri*ng *C*omplex (TRiC) accomplishes this task by providing a folding chamber for the substrate that is regulated by an Adenosine triphosphate (ATP) hydrolysis-dependent cycle. Once delivered to and recognized by TRiC, the nascent substrate enters the folding chamber and undergoes folding and release in a stepwise manner. During the process, TRiC subunits and cochaperones such as prefoldin and phosducin-like proteins interact with the substrate to assist the overall folding process in a substrate-specific manner. Coevolution between the components is supposed to consult the binding specificity and ultimately expand the substrate repertoire assisted by the chaperone network. This review describes the TRiC chaperonin and the substrate folding process guided by the TRiC network in cooperation with cochaperones, specifically focusing on recent progress in structural analyses.

## INTRODUCTION

The process of protein folding in living organisms remains a central enigma in the field of biology. The expanding catalog of diseases associated with protein misfolding, encompassing conditions from cancer to neurodegenerative disorders, underscores the critical nature of this inquiry into human health (Boudiaf-Benmammar et al., 2013, Scott and Frydman, 2003, Sweeney et al., 2017, Todd and Lim, 2013). Molecular chaperones belonging to various classes play pivotal roles in facilitating the maturation of freshly synthesized polypeptides. While certain chaperones, such as nascent polypeptide-associated complex (NAC) or Heat shock protein (Hsp) 70, assist in the folding of a broad spectrum of proteins, others, typified by ring-shaped chaperonins, are specialized in shepherding a specific subset of proteins that constitute the proteome (Hartl and Hayer-Hartl, 2002, Koplin et al., 2010, Willison, 2018). Intriguingly, the proteins that rely on *T*ailless complex polypeptide 1-*Ri*ng *C*omplex (TRiC) for their folding, many of which are indispensable for the viability of an organism, stand out due to their propensity for aggregation and their intricate topological structures (Brehme et al., 2014, Nollen et al., 2004).

Group I chaperonins are prevalent in bacterial cytosols and eukaryotic organelles derived from endosymbiosis (Cheng et al., 1990, Hemmingsen et al., 1988). These chaperonin systems are occasionally found in archaea as well (Braig et al., 1995, Conway de Macario et al., 2003). The group I chaperonin assembly comprises 2 essential components: a tetradecameric Hsp60 and a heptameric co-chaperone Hsp10. In the case of *Escherichia coli*, the Hsp60 component, known as GroEL, forms 2 7-fold symmetric rings connected by a 2-fold inter-ring symmetry axis (Braig et al., 1995). There is a central cavity in each GroEL ring where client proteins are encapsulated for the folding process. The Hsp10 cochaperone, called GroES in *E. coli*, binds to GroEL in an ATP-dependent manner, effectively acting as a “lid” to obstruct substrate escape while significantly expanding the size of the folding chamber (Bukau and Horwich, 1998, Xu et al., 1997). In contrast, group II chaperonins, are primarily found in archaea and the eukaryotic cytosol. These chaperonins also comprise 2o stacked rings, each consisting of 8 subunits with a molecular weight ranging from 50 to 60 kDa (Cong et al., 2010, Frydman et al., 1992, Kim et al., 2002, Leitner et al., 2012, Lopez et al., 2015). However, they lack an obligatory cochaperone akin to the group I chaperonins. Instead, they possess an inherent lid mechanism that closes the folding chamber, allowing them to fold substrates in vitro without necessitating accessory proteins (Gestaut et al., 2019, Zhang et al., 2010). Eukaryotic *C*haperonin *C*ontaining *T*ailless complex polypeptide 1 (CCT)/TRiC chaperonin is one of the group II chaperonins, whose name is derived from CCT or TRiC (Frydman et al., 1992, Kubota et al., 1994, Silver et al., 1979). Of note, it is crucial to recognize that TRiC, as well as another group II chaperonins, does not operate alone in the cell. On the contrary, they appear to be central components of an intricate network of co-chaperones (Balchin et al., 2016). An illustrative example is the hexameric prefoldin (PFD) complex, which is often believed to bind to unfolded substrates and prevent their aggregation before transferring them to the chaperonin (Gestaut et al., 2019, Vainberg et al., 1998). Additionally, the phosducin-like proteins (PhLPs) have been demonstrated to enhance the TRiC-mediated folding of several substrates (Stirling et al., 2007, Willardson and Howlett, 2007).

The role of TRiC/CCT in the folding process is especially interesting due to its involvement in assisting the folding of numerous eukaryotic proteins that exhibit a unique challenge. These proteins cannot fold spontaneously or with the aid of any other chaperone, regardless of the conditions employed (Balchin et al., 2018, Gao et al., 1993). While various chaperones can bind to proteins such as actin and tubulin and prevent their aggregation, only TRiC can facilitate their accurate folding (Balchin et al., 2018, Sternlicht et al., 1993, Yaffe et al., 1992). The existence of TRiC obligatory substrates, as well as large proteome assisted, draws an explanation of the folding pathway using the essential chemical and topological environment provided by the TRiC network, involving a potential co-evolutionary relationship between TRiC and selected eukaryotic proteins (Llorca et al., 1998, Spiess et al., 2006, Stein et al., 2019, Yam et al., 2008).

### Structure and ATP-driven Asymmetric Conformational Changes of TRiC Complex

**Fig. 1:**
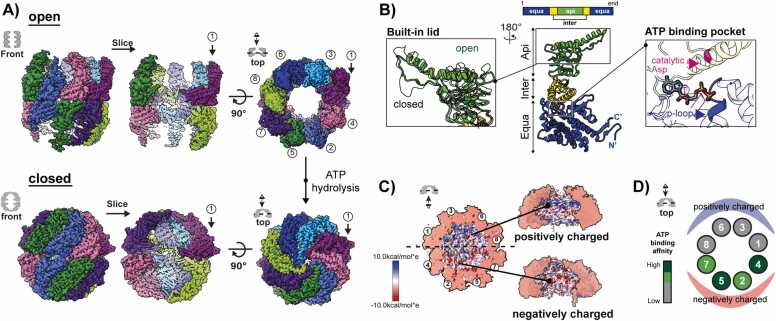
Structure of TRiC and ATP-driven structural change. (A) The cryo-EM structure of TRiC in either open or closed state, and chamber closing driven by ATP hydrolysis. Structure of open TRiC: EMD-33053, structure of closed TRiC: EMD-32926 (Liu et al., 2023). (B) CCT subunit with three domains with a zoom-in view of the built-in lid in the apical domain and ATP binding pocket in the equatorial domain. (C) Asymmetric charge distribution on the inner chamber wall of the closed TRiC. Upon chamber closing, half of the hemisphere of CCT1/3/6/8/ is charged positively, while the other half hemisphere is charged negatively. Figures are adopted from Park et al. (2023)*.* (D) Color-coded diagram of different ATP affinities of individual CCT subunits. Deep green: high ATP binding affinity, chartreuse: mild ATP binding affinity, gray: low binding affinity. Figures are modified from Gestaut et al. (2019a)*.*

TRiC is a 1 MDa complex consisting of 8 paralogous subunits, CCT1-8, that assemble into a double-ring hexadecane structure (Fig. 1A) (Frydman et al., 1992, Leitner et al., 2012, Lin and Sherman, 1997). Each subunit has a distinctive 3-domain architecture. This architecture includes an apical domain positioned at the apex housing both the substrate recognition site and the lid-forming loop. The equatorial domain accommodates the ATP-binding site and serves as an assembly platform. The intermediate domain communicates ATP cycling from the equatorial domain to drive movements in the apical domain (Fig. 1B) (Balchin et al., 2018, Joachimiak et al., 2014, Llorca et al., 1998, Spiess et al., 2006).

The structure reveals asymmetry in various functional aspects, and each ring maintains an identical subunit order (CCT 2-4-1-3-6-8-7-5) with a 2-fold symmetry axis (Leitner et al., 2012). ATP binding to TRiC initiates the formation of specific contacts in the apical domains and generates a more compact and rigid open conformation (Meyer et al., 2003). Subsequent ATP hydrolysis induces a conformational rotation of the apical domains. This rotation accomplishes 2 critical tasks: it closes the lid and releases the bound substrate into the central chamber, which now adopts a highly polar/charged environment (Fig. 1A). In contrast to archaeal group II chaperonin, built-in lids of CCT5-2-4 and CCT3-6-8 create negative and positive hemispheres upon chamber closure, respectively, which are highly conserved and essential for TRiC function (Fig. 1C) (Leitner et al., 2012). Single-molecule studies have revealed TRiC binding to around 7 or 8 nucleotides at nearly all ATP concentrations, potentially indicating uneven occupancy of ATP pockets on each subunit. ATP titration has also uncovered a hierarchy of ATP affinity, with CCT4/5 > CCT1/2 >>> CCT7/8/6/3 (Fig. 1D) (Reissmann et al., 2012). Consequently, the asymmetric use of ATP by TRiC has given rise to a model suggesting sequential closure, commencing with the high-affinity hemisphere and progressing through the other subunits. Early structural investigations have detected varying degrees of ring closure based on ATP concentrations. Subsequent structural work employing Hydrogen Deuterium exchange Mass Spectrometry (H/D exchange MS) and cryo-Electron Microscopy (cryo-EM) lends support to the notion that the ATP-dependent conformational change originates in the “high” ATP affinity lobe of TRiC (CCT5/2/4/1) (Balchin et al., 2018, Gestaut et al., 2019, Zang et al., 2016).

### Substrate Recognition by and Delivery to TRiC

#### Substrate Recognition by TRiC

**Fig. 2:**
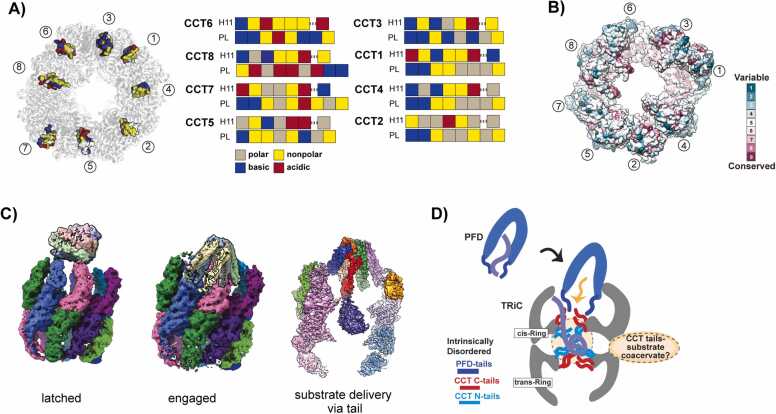
Characteristics of TRiC that convey substrate recognition and delivery. (A) Charge distribution in the apical domain of each CCT subunit, labeled as a color code; gray: polar, yellow: nonpolar, blue: basic, red: acidic. Each box represents one helix. (B) Diagram of conservation score of each CCT subunit. The surface is colored from calculated scores using ConSurf (Ashkenazy et al., 2016). (C) Structures of PFD-TRiC complex visualized by cryo-EM. Left: latch-binding mode of PFD on TRiC (EMD-0493), middle: “engaged” binding mode of PFD on TRiC, whose tail is deep inside the open chamber (EMD-0491), right: PFD-TRiC complex during substrate delivery, whose tails of PFD are deep inside the open chamber. Substrate-induced density resides in the inter-ring chamber of the open TRiC (EMD-32823). (D) Schematic diagram of a model of substrate delivery mediated by PFD to TRiC. When a nascent substrate is bound to PFD, it delivers the substrate into TRiC by using its extended tail. Unstructured, charged N-tail and C-tail of CCT subunits contribute to taking over the substrate, allowing the substrate located in the inter-ring space. Figures are adopted from Gestaut et al. (2023)*.*

The principles governing substrate recognition by TRiC remain a subject of limited comprehension. In vivo, TRiC is responsible for folding a specific group of cellular proteins, implying its certain degree of specificity. However, the substrates also accommodate various functions and structures, suggesting the potential of the chaperonin to cover a wide spectrum of proteins. The prevailing hypothesis posits that the apical domains of individual TRiC subunits recognize distinct motifs in substrates (Joachimiak et al., 2014). The distinctive patterns of polar and hydrophobic residues in each CCT subunit dictate their unique substrate binding capabilities within the complex (Fig. 2A). This diversification of TRiC subunits furnishes a modular array of binding specificities, facilitating the combinatorial recognition of substrate polypeptides (Spiess et al., 2006, Yam et al., 2008). This distinctive feature likely underpins TRiC's exceptional aptitude for folding structurally diverse and intricately configured substrates. Additionally, evolutionary analyses suggest that the diversification of TRiC subunits, which differentiate it from its simpler archaeal ancestors, played a pivotal role in enabling eukaryotic genomes to acquire proteins with novel folds and functions (Fig. 2B) (Joachimiak et al., 2014).

(A) Adopted from Gestaut et al. (2019a). (B) Adopted from Park et al. (2023).

#### Cochaperone-mediated Substrate Delivery to TRiC

PFD, also known as GimC, is a superfamily of proteins integral to protein folding complexes (Vainberg et al., 1998). This molecular entity is categorized as a heterohexameric molecular chaperone and is present in both archaea and eukarya, including the human system (Leroux et al., 1999, Siegert et al., 2000). The PFD molecule acts as a mediator working in tandem with a TRiC chaperonin to assemble into a chaperone complex (Gestaut et al., 2019b). This complex has the crucial function of accurately folding other nascent proteins (Gestaut et al., 2019, Gestaut et al., 2023, Hansen et al., 1999). The interaction of PFD with TRiC hinges on a conserved electrostatic interface as a pivotal point in this process. It exhibits dynamic behavior, shifting between an open “latched” conformation and an “engaged” conformation, and precisely aligning the substrate binding chambers of both TRiC and PFD (Fig. 2C) (Gestaut et al., 2019b). The substantial impact of PFD on the TRiC-actin complex significantly increases both the yield and velocity of the folding reaction (Gestaut et al., 2019b). In the context of PFD-bound β-tubulin, its introduction into the open TRiC chamber results in interactions with the disordered CCT tails, giving rise to a compact entity devoid of recognizable folded structures (Fig. 2C) (Gestaut et al., 2019, Gestaut et al., 2023). These disordered tails, confined in the TRiC chamber and acting like a tethered solvent, preserve the fluidity of the substrate and foster a dynamic yet compacted state. This liquid-like coacervate characteristic may serve as a safeguard against the formation of unproductive or trapped intermediates, thereby priming the substrate for efficient folding during the conformational changes of TRiC (Fig. 2D).

### Chamber Closure Triggers Substrate Folding

Many studies have attempted to determine the whereabouts of the substrate in the subsequent phase of the folding cycle, triggered by ATP hydrolysis and the chamber closure of TRiC, using biophysics or biochemistry (Balchin et al., 2018, Neirynck et al., 2006, Rüßmann et al., 2012, Stuart et al., 2011). However, structural visualization of the substrate in the chamber has remained elusive due to its innate heterogeneity. In light of the cryo-EM method and the power of heterogeneity analysis, multiple studies have recently succeeded in capturing snapshots of the folded or partially folded substrate in the TRiC folding chamber in the presence of ATP-Aluminium fluoride (AlFx).

**Fig. 3:**
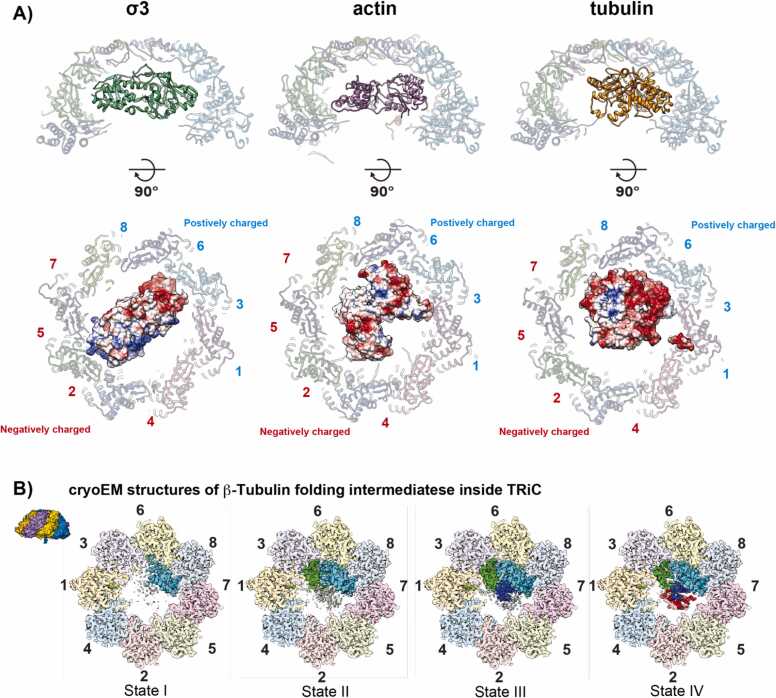
Substrate specific interaction in the TRIC folding chamber. (A) Three structures of substrates encapsulated in closed TRiC (σ3, tubulin, actin, PDB: 7LUP, 7TUB, 7NVM, respectively) shown in cartoon models at side slice view, and electrostatic charge surface of substrates encapsulated at top slice view. Each substrate shows specific interactions with different CCT subunits. (B) cryo-EM structures of 4 folding intermediates, showing progressive folding of the substrate guided by TRiC. Figures are adopted from Gestaut et al. (2023)*.*

Among TRiC's substrates, σ3, tubulin, and actin in the closed TRiC are visualized by using cryo-EM, either in the yeast system or human system (Fig. 3A). The first resolved structure is one of the reovirus outer capsid proteins, σ3 (Guo et al., 2021, Knowlton et al., 2021). The substrate shows a near-native conformation and is oriented inside the chamber, stretching across the asymmetrically charged hemisphere with charge complementarity, whose positively charged surface interacts with a negatively charged inner wall of the TRiC chamber, and vice versa. Similarly, tubulin possesses a native conformation in both yeast and human TRiC and has strong interactions with the positively charged patches of TRiC, while no interaction is observed with the negatively charged half-hemisphere of a folding chamber (Gestaut et al., 2023, Han et al., 2023, Kelly et al., 2022, Liu et al., 2023). Meanwhile, actin exhibits electrostatic interactions with both hemispheres. Interestingly, actin has a different conformation compared to G-actin, which shows a “much opened” conformation, with a wider angle between the 2 wings of actin (Kelly et al., 2022). This suggests that TRiC can alter the structure of the substrate inside its chamber during or after folding, either in direct or indirect ways. Interestingly, σ3, tubulin, and actin all have strong surface charges complementary to the inner wall of the closed TRiC hemisphere. This suggests that TRiC may interact strongly with the substrate through electrostatic interaction and that the charge division acts differently depending on the substrate. In addition, recent studies also report the interaction between the tails of CCT subunits and substrates, suggesting a much-diversified way of interaction adopted by the TRiC complex (Gestaut et al., 2023, Liu et al., 2023).

Another breakthrough came with the attempt to visualize the substrate folding process in the TRiC chamber using cryo-EM, which revealed 4 distinct β-tubulin conformations inside the chaperonin (Fig. 3C) (Gestaut et al., 2023). These 4 structures have densities connecting various secondary structure elements, indicative of 4 progressively folded β-tubulin intermediates residing within the closed TRiC chamber. These 4 structural states of β-tubulin in the chamber of TRiC suggest a folding pathway where discontinuous sequence elements in the encapsulated β-tubulin polypeptide fold progressively in close association with the inner chaperonin chamber, assembled into specific domains to reach the native state. This strategic folding process ensures that the surface of the folded N-domain and C-domain securely anchors to the TRiC chamber wall, not only establishing a native topological arrangement but also orienting the growing folding intermediates in such a way that their hydrophobic core extends inward, inside the chamber. In this manner, the interactions between TRiC and tubulin meticulously shape the tubulin folding landscape and prevent the formation of trapped folding intermediates along the way.

### Release or Retention of a Folded Substrate by TRiC

Once TRiC substrates achieve their desired and native conformations, the chaperonin must orchestrate their release to enable them to fulfill their biological functions. TRiC releases its cargo upon ATP hydrolysis-induced conformational shifts (Gestaut et al., 2019a). These shifts instigate modifications in the interaction dynamics between TRiC and its substrate according to the conformational state of the substrate (Gestaut et al., 2023). Typically, the tightly embraced, properly folded protein should experience an energetically favorable departure from the TRiC chamber. The specifics of this release mechanism, whether it ensues by passive diffusion, driven by the intrinsic motion of the protein, or through active assistance, can depend on cooperation with the conformational state of TRiC. However, it is crucial to recognize that certain substrates remain ensconced in TRiC even after successful folding (Gao et al., 1993). Notably, proteins such as the Von Hippel-Lindau disease-related protein necessitate TRiC's guidance for proper folding but exhibit a reluctance to spontaneously disengage from TRiC's grasp (Melville et al., 2003). These substrates only achieve liberation when they can establish binding interactions with specific cofactors, such as elongin BC in the case of the Von Hippel-Lindau disease-related protein (Feldman et al., 1999). There is a similar scenario with Acute Myeloie Leukemia 1 (AML1), which is proposed as a TRiC substrate but necessitates the presence of core-binding factor beta for its eventual release (Roh et al., 2016, Roh et al., 2016). After the substrate is properly folded and released, the proteins can participate in many complicated biological phenomena, either in association with other partners or through additional modifications (Ahn et al., 2023, Shao et al., 2023). In this capacity, TRiC operates not only as a chaperone but also as a holdase, shielding substrates to prevent undesired activities until the appropriate conditions for their release are met. While the release of folded substrates from TRiC is a pivotal event in chaperonin-mediated folding, the mechanisms governing this step remain significantly understudied.

### TRiC and Cochaperone Cooperate to Support Proteomic Complexities

**Fig. 4:**
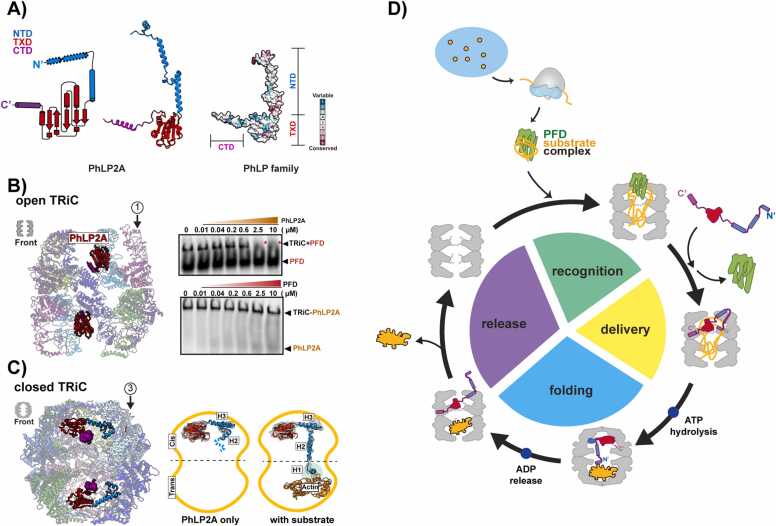
TRiC-cochaperone network to support proteomic complexities. (A) Domain conservation of PhLP family. Left: topology diagram and Alphafold-predicted model of PhLP2A, right: residue-level conservation score of human PhLPs. (B) Left: cryo-EM structure of open TRiC-PhLP2A complex and right: modulation of PhLP2A on TRiC-bound PFD. The addition of PhLP2A can displace PFD bound on TRiC, but not vice versa. (C) Left: structure of closed TRiC-PhLP2A complex visualized by cryo-EM and right: participation of PhLP2A in substrate folding. When the substrate is encapsulated in the opposite chamber, N-terminal helices of PhLP2A extend and directly bind to the hydrophobic groove on actin. (D) Proposed mechanism of TRiC-cochaperone network to support protein folding. After translation, PFD receives the nascent substrate and delivers it to TRiC. When it is recognized by the apical domain of CCT, the substrate is delivered inside TRiC. ATP cycle-dependent chamber closing and reopening allow the substrate folding and release, respectively. During the substrate folding cycle, another cochaperone, such as PhLP2A, orchestrates with TRiC to modulate other cochaperones, or assists substrate folding. Figures are modified and adopted from Park et al. (2023)*.*

TRiC folds approximately 10% of the cytoplasmic proteome and employs a variety of strategies to support the folding of various substrates (Yam et al., 2008). The hetero-oligomeric nature of TRiC is key to recognizing distinct motifs in its various substrates and promoting their folding in the enclosed chamber (Balchin et al., 2018, Joachimiak et al., 2014). On the other hand, TRiC collaborates with several cochaperones, including PFD and PhLPs (Gestaut et al., 2019, Han et al., 2023, Kelly et al., 2022, McLaughlin et al., 2002). Notably, the PhLP family consists of approximately 30 kDa cytosolic proteins that also participate in the regulation of TRiC-mediated protein folding (Blaauw et al., 2003, Miles et al., 1993, Savage et al., 2000). These PhLP homologs share a common domain structure featuring a central thioredoxin-like domain (TXD) flanked by variable-length, flexible N-terminal domain and C-terminal domain (NTD and CTD, respectively) (Fig. 4A) (Park et al., 2023). Each PhLP isoform exhibits unique activity and specificity toward different TRiC substrates. The isoforms share conserved TRiC binding components while evolving distinct substrate-binding elements that dictate their roles in the TRiC folding environment (Park et al., 2023). PhLPs are exclusive to eukaryotes, and their emergence may correlate with the rise in proteomic complexity. The combined use of PhLPs as substrate-specific cochaperones amplifies the capacity of TRiC to fold various substrates. Recent studies have revealed the domain-specific connections between PhLP2A and TRiC, and various functions of the cochaperone in the TRiC network (Han et al., 2023, Kelly et al., 2022, Park et al., 2024).

#### PhLP2A Binds to the Open TRiC Chamber and Modulates TRiC-PFD Interactions

In a recent cryoEM investigation, the interaction between PhLP2A or its yeast homolog plp2 and open TRiC was revealed (Fig. 4B) (Han et al., 2023, Park et al., 2024). PhLP2A adopts an extended conformation in the open TRiC chamber, with its domains targeting distinct subunit-specific sites in the chaperonin. The central PhLP2A TXD domain becomes enclosed in the TRiC chamber, while the amphipathic helix of CTD forms a hydrophobic zipper motif with the equatorial domain of CCTs. The NTD of PhLP2A can adopt 2 orientations that interact with apical regions of either CCT3 or CCT4, which is also the binding site for another cochaperone PFD. Further structural and biochemical studies also reveal that their binding to TRiC is mutually exclusive, and PhLP2A can displace the PFD bound to TRiC, but not vice versa (Park et al., 2023). Collectively, these phenomena suggest a role for PhLP2A in orchestrating PFD and TRiC association and establishing a directional TRiC cochaperone network. The link between these findings and the role of PhLPs in TRiC-mediated folding awaits further exploration.

#### ATP-induced TRiC Closure Folds the Substrate in Collaboration With PhLP2A

TRiC collaborates harmoniously with PhLPs in a concerted mechanism to aid substrate folding. Upon ATP hydrolysis-driven chamber closing, TRiC undergoes alterations in interaction interfaces inside, which triggers the repositioning and reconformation of PhLP2A through domain-specific contacts involving TRiC components (Fig. 4C) (Kelly et al., 2022, Park et al., 2024). As a result, the transformation causes a significant movement of negatively charged NTD of PhLP2A to be compacted and encapsulated inside the TRiC, and attached to the positively charged inner wall of CCT3/CCT6 intermediate domains. Furthermore, the TXD domain rotates about 180° and shifts its contacts from the equatorial domains of CCT subunits to the apical CCT5/2/4 lid segments. Importantly, with substrate actin in the opposite chamber, PhLP2A experiences further conformational changes, particularly in H1 and H2 of NTD (Fig. 4C) (Kelly et al., 2022, Park et al., 2024). This reorientation enables H2 to traverse the chamber, positioning H1 for direct binding to the substrate and concealing a hydrophobic groove surrounding 2 actin helices. This interaction is supposed to stabilize the exposed hydrophobic core of actin intermediates during substrate folding. Notably, mutations in actin residues corresponding to PhLP2A's H1 binding site significantly affect actin folding (Rommelaere et al., 2003). The intricate interplay between TRiC and PhLPs orchestrates a hydrophobic collapse that propels the substrate folding process. Collectively, these studies suggest that PhLP2A has bifunctional modules and works as a “chaperone-in-chaperonin” during the folding cycle (Fig. 4D).

## SUMMARY

The eukaryotic chaperonin TRiC is required to fold many proteins that are indispensable for survival (Balchin et al., 2016, Gestaut et al., 2019). During the folding process assisted by TRiC and cooperating cochaperones, substrates undergo 4 major steps: recognition, delivery, folding, and release (Gestaut et al., 2019, Spiess et al., 2006). In this review, TRiC chaperonin and the folding processes are described regarding recent progress in structural analyses. TRiC experiences ATP-driven cycling with a variety of CCT subunits, which appears to contribute to expanding the substrate repertoire (Spiess et al., 2006). The following substrate folding processes are described with recently visualized cryo-EM structures, which show the cooperation between cochaperones and TRiC or visualize folding intermediates on the protein folding pathway (Gestaut et al., 2023, Han et al., 2023, Kelly et al., 2022, Knowlton et al., 2021). Finally, we cover PhLP2A which was recently reported cochaperone that cooperates with the TRiC network in a bifunctional manner (Park et al., 2023). Further investigation on the orchestration between TRiC, cochaperone, and substrate will provide insights into the protein folding mechanism from the viewpoint of protein evolution.

## Author Contributions

H.K. and J.P. conceptualized the study and wrote the manuscript. S.-H.R. conceptualized the study, wrote the manuscript, and supervised the project.

## Declaration of Competing Interests

The authors declare no competing interests.
